# Healthcare professionals, substance use and adverse childhood experiences

**DOI:** 10.1186/1940-0640-10-S2-O3

**Published:** 2015-09-24

**Authors:** Agnieszka Baklazec, Elizabeth Pace

**Affiliations:** 1 Peer Assistance Services, Inc., Denver, USA

## Background

Adverse Childhood Experiences (ACE) are a predictor to medical and mental health conditions, including substance use disorders. The ACE study, conducted by Kaiser Permanente and Center for Disease Control and Prevention, links childhood trauma to health and social consequences.[1,2] Peer Assistance Services, Inc., a Colorado nonprofit organization, implements Peer Health Assistance Programs (PHAP), for licensed healthcare professionals who have substance use and/or mental health disorders affecting public safety.

The objective is to better understand the characteristics of clients living with substance use and/or mental health conditions, and to improve outcomes, PHAP identified characteristics of clients with an ACE score of 4 or higher. The likelihood of clients underreporting ACE scores was also studied.

## Material and methods

The ACE questionnaire is administered to clients and a clinical ACE score is given by the clinician. The score assigns one point for each category of exposure to child abuse and/or neglect. Points are summed for a score from 0 to 10. The higher the score, the greater the exposure.[3] The PHAP hypothesized clients with higher ACE scores would be more likely to have a substance use or mental health diagnoses, evidenced by increased score on the AUDIT, DAST-10 or PHQ-9.

## Results

Preliminary analysis of 171 clients shows that 78 % with an ACE score of 4 or higher have a substance use and/or mental health condition. The ACE score did not always correlate with increased scores on the AUDIT, DAST-10 or PHQ-9. Also, 76% of clients aware of their trauma history had the same ACE score as the clinician's ACE score, indicating that 24% of PHAP clients underreported their ACE score.

## Conclusions

The data suggests ACE have an enormous impact on individuals and their careers. Screening for ACE in PHAPs, in addition to AUDIT, DAST-10 or PHQ-9, may impact long term recovery and safe return to practice.
